# Surveillance of the major pathogenic arboviruses of public health concern in Gabon, Central Africa: increased risk of West Nile virus and dengue virus infections

**DOI:** 10.1186/s12879-021-05960-9

**Published:** 2021-03-17

**Authors:** Yuri Ushijima, Haruka Abe, Georgelin Nguema Ondo, Rodrigue Bikangui, Marguerite Massinga Loembé, Vahid R. Zadeh, Joseph G. E. Essimengane, Armel V. N. Mbouna, Emmanuel B. Bache, Selidji T. Agnandji, Bertrand Lell, Jiro Yasuda

**Affiliations:** 1 Department of Emerging Infectious Diseases, Institute of Tropical Medicine (NEKKEN), Nagasaki University, Nagasaki, Japan; 2 Centre de Recherches Médicales de Lambaréné, Lambaréné, Gabon; 3 Institute for Tropical Medicine, University of Tübingen, Tübingen, Germany; 4 African Society for Laboratory Medicine, Addis Ababa, Ethiopia; 5 Africa Centres for Disease Control and Prevention, Johannesburg, South Africa; 6 Graduate School of Biomedical Sciences, Nagasaki University, Nagasaki, Japan; 7 Université des Sciences et Techniques de Masuku, Franceville, Gabon; 8 Division of Infectious Diseases and Tropical Medicine, Medical University of Vienna, Vienna, Austria; 9 National Research Center for the Control and Prevention of Infectious Diseases (CCPID), Nagasaki University, Nagasaki, Japan

**Keywords:** Surveillance, Arboviruses, West Nile virus, Dengue virus, Gabon, Africa

## Abstract

**Background:**

Increasing arbovirus infections have been a global burden in recent decades. Many countries have experienced the periodic emergence of arbovirus diseases. However, information on the prevalence of arboviruses is largely unknown or infrequently updated because of the lack of surveillance studies, especially in Africa.

**Methods:**

A surveillance study was conducted in Gabon, Central Africa, on arboviruses, which are a major public health concern in Africa, including: West Nile virus (WNV), dengue virus (DENV), Zika virus (ZIKV), yellow fever virus (YFV), chikungunya virus (CHIKV), and Rift Valley fever virus (RVFV). Serological and molecular assays were performed to investigate past infection history and the current status of infection, using serum samples collected from healthy individuals and febrile patients, respectively.

**Results:**

The overall seroprevalence during 2014˗2017 was estimated to be 25.3% for WNV, 20.4% for DENV, 40.3% for ZIKV, 60.7% for YFV, 61.2% for CHIKV, and 14.3% for RVFV. No significant differences were found in the seroprevalence of any of the viruses between the male and female populations. However, a focus on the mean age in each arbovirus-seropositive individual showed a significantly younger age in WNV- and DENV-seropositive individuals than in CHIKV-seropositive individuals, indicating that WNV and DENV caused a relatively recent epidemic in the region, whereas CHIKV had actively circulated before. Of note, this indication was supported by the detection of both WNV and DENV genomes in serum samples collected from febrile patients after 2016.

**Conclusions:**

This study revealed the recent re-emergence of WNV and DENV in Gabon as well as the latest seroprevalence state of the major arboviruses, which indicated the different potential risks of virus infections and virus-specific circulation patterns. This information will be helpful for public health organizations and will enable a rapid response towards these arbovirus infections, thereby preventing future spread in the country.

**Supplementary Information:**

The online version contains supplementary material available at 10.1186/s12879-021-05960-9.

## Background

Arboviruses are transmitted by arthropod vectors such as mosquitos, ticks, sandflies, and midges [1]. With the geographic expansion of vector habitats and an increasing impact on susceptible populations, arboviruses have become emerging or re-emerging pathogens. Several arboviruses have been a significant threat to public health worldwide in recent decades. Dengue virus (DENV) infection is now estimated to be the most common arboviral infection globally, putting 3.9 billion people at risk per year and occurring in at least 128 countries [2, 3]. Zika virus (ZIKV) has attracted global attention since an outbreak in Brazil in 2015, owing to its rapid spread to other countries and possible relationship with severe birth defects caused by infection with the virus during pregnancy [4]. West Nile virus (WNV) can cause neurological diseases, and the virus has spread and become widely established in North America and Europe since its introduction into New York State in 1999 [5]. Despite the availability of an effective vaccine, the re-emergence of infections with the yellow fever virus (YFV), has been continually reported in tropical countries, including Brazil, Angola, the Republic of the Congo, and the Domestic Republic of the Congo, [6, 7].

The difficulty in diagnosing ongoing arbovirus diseases is closely related to asymptomatic infections or even nonspecific signs and symptoms in majority of the patients infected with arboviruses (e.g., approximately 70% for DENV and 60% for ZIKV), leading to unrecognised infection cases. Moreover, patients might be misdiagnosed with other febrile illnesses such as malaria [1, 8–10]. These subclinical infections with arboviruses can cause underreporting of cases and the silent transmission of arbovirus such as DENV [11, 12]. Undoubtedly, molecular methods are useful for accurately diagnosing patients presenting with suspected symptoms, while serological investigations are also informative in understanding the history of arbovirus infections, independent of signs and symptoms.

Many countries in Africa, where most of the known arboviruses have been detected, have experienced the periodic emergence of arbovirus diseases [1, 9, 10]. However, information on the prevalence of arbovirus diseases is largely unknown or infrequently updated due to the lack of surveillance studies in Africa. Gabon, a Central African country, is expected to be an arbovirus disease endemic country; with its tropical rainforest climate, it is a preferred habitat for arthropod vectors [1]. There are several reports on the emergence of arbovirus infections in Gabon. Two large outbreaks of dengue with DENV serotype 2 (DENV-2) occurred in 2007 and 2010, with simultaneous chikungunya virus (CHIKV) co-infections, reported in humans and mosquitoes [13–16]. Moreover, DENV serotype 3 (DENV-3) was reported to have been circulating continuously in the country since 2010 [17]. The ZIKV genome was also detected in both humans and mosquitos in the country in 2007 [17]. However, available information on arbovirus infections is still limited, leading to a non-recognition of their risks and a delayed response to prevention.

In this study, we conducted a surveillance study in Gabon from 2014 to 2020, with samples of healthy individuals and febrile patients, aiming to investigate their past infection histories and current status of infection with the major pathogenic arboviruses of public health concern in Gabon, Central Africa: WNV, DENV, ZIKV, YFV, CHIKV, and Rift Valley fever virus (RVFV).

## Methods

### Study ethics

The study was approved by the Institutional Review Boards of Centre de Recherches Médicales de Lambaréné (CERMEL), the National Ethical Committee in Gabon, and Nagasaki University (approval numbers. CEI-007, N̊_0080/2019/PR/SG/CNER, and 170,921,177, respectively). Written informed consent was obtained from all the participants or their parents.

### Study population

**Fig. 1:**
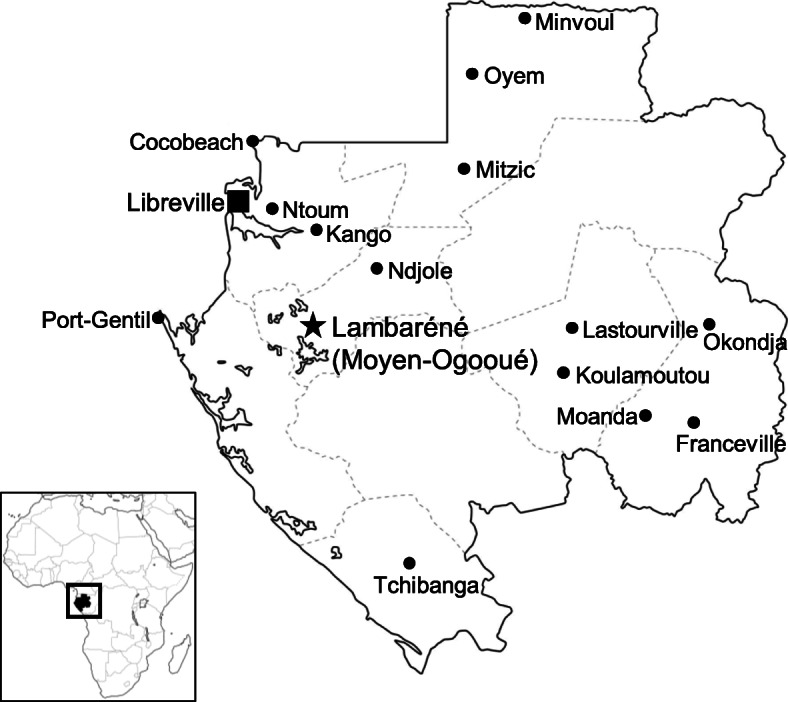
Location of the study area, Lambaréné, and major cities in Gabon. Black star: Lambaréné; Black square: the capital city; Black circle: other major cities mentioned in the main text and Additional file 3. The dotted line indicates each province in the country

For the serological assays, 462 serum samples were collected from healthy individuals who visited CERMEL, Lambaréné, the capital of the Moyen-Ogooué province in Gabon (Fig. 1), between November 2014 and January 2017. For the molecular assays, 1189 serum samples were collected from febrile patients (body temperature ≥ 37.5 °C) who visited the Albert Schweitzer Hospital in Lambaréné and CERMEL between January 2015 and March 2020 (samples were unavailable during 2018–2019). All samples were collected as a part of a project for establishing a surveillance system for viral diseases which cause fever. In this study, the participants’ residence was in and near Lambaréné and their age was restricted to ≥1 year. Demographic information (age and sex) of the participants was also collected.

### Enzyme-linked immunosorbent assay (ELISA)

All samples were tested for IgG antibodies using an in-house indirect ELISA. Recombinant proteins for the following arboviruses were obtained commercially: non-structural protein 1 (NS1) for WNV (Native Antigen, Oxford, UK), DENV-1 (Enzo Biochem), DENV-2 (Enzo Biochem), DENV-3 (Native Antigen), DENV-4 (Native Antigen), ZIKV (Native Antigen), and YFV (Enzo Biochem, Farmingdale, NY, USA); envelope protein 1 (E1) for CHIKV (Native Antigen); and nucleoprotein (NP) for RVFV (ViroQuest Corporation, Osaka, Japan). For the detection of IgG antibodies to DENV, mixtures of NS1, antigens from four different serotypes were used as antigens.

The MaxiSorp ELISA plates (Thermo Fisher Scientific, Waltham, MA, USA) were coated with 100 ng of viral antigens in 100 μL phosphate-buffered saline (PBS) per well and incubated overnight at 4 °C. The control wells were incubated without the antigens. The wells were then blocked with 100 μL of 5% skim milk in PBS containing 0.05% Tween 20 (PBS-T) for 1 h at 23–25 °C. After incubation, the wells were washed three times with PBS-T. The serum and plasma samples were diluted 1:100 in PBS-T containing 2% skim milk and added to a final volume of 100 μL per well. The plates were incubated for 1 h at 37 °C, and then washed three times. One hundred μL of a 1:50,000 dilution of horseradish peroxidase-conjugated goat anti-human IgG antibody (Bethyl Laboratories, Montgomery, TX, USA) was added and incubated for 1 h between 23 and 25 °C. After washing five times, 100 μL of 3,3′,5,5′-tetramethylbenzidine (TMB) substrate (Thermo Fisher Scientific) was added and followed by 100 μL of 0.2 M sulfuric acid. The optical density (OD) was measured at 450 nm using a FilterMax F5 microplate reader (Molecular Devices, San Jose, CA, USA). For each sample, we calculated the OD value of the antigen-coated well and subtracted the value of the corresponding control well from it to obtain the adjusted OD value. The cut-off value for each virus was set as (mean + 3 standard deviation of OD values from five negative samples). A sample was considered seropositive when its adjusted OD value was greater than the cut-off value.

### Cross-reactivity within flaviviruses

To determine the cross-reactivity against each flavivirus NS1 antigen used in this study, the following types of serum and plasma were used as anti-flavivirus positive controls: anti-WNV plasma obtained commercially (SeraCare Life Sciences, Milford, MA, USA); anti-DENV, ZIKV, and CHIKV sera kindly provided by Dr. Kouichi Morita, and anti-YFV serum collected from a YFV-vaccinated volunteer. Briefly, a two-fold dilution series of the anti-flavivirus positive controls from the 100-fold dilution was applied for all flavivirus NS1-based ELISAs to find dilution rates with positive control OD values of ≈ 1.5. Again, the OD value adjusted anti-flavivirus positive controls were used for the ELISA to determine cross-reactivity. A positive control was defined on the basis of cross-reactivity against the indicated antigen when its OD value was greater than the cut-off value. Arbovirus seropositivity in healthy individuals in Gabon was determined using the criteria described below (Additional file 1: Table S1). In summary, the WNV NS1 antigen showed cross-reactivity with an anti-ZIKV positive control and the DENV NS1 antigen with both anti-ZIKV and anti-YFV positive controls. Neither ZIKV NS1 nor YFV NS1 antigens showed cross-reactivity with the other anti-flavivirus positive controls. Accordingly, when a sample was double-positive for WNV NS1 and ZIKV NS1 antigens, the sample was considered to be seropositive for ZIKV, but not for WNV. Similarly, when the sample was double-positive for DENV NS1 with either ZIKV NS1 or YFV NS1 antigens, the sample was considered seropositive for either ZIKV or YFV, but not for DENV. In the case of a sample positive for ZIKV NS1, YFV NS1, CHIKV (the genus *Alphavirus*) E1 or RVFV (the genus *Phlebovirus*) NP antigens, the sample was considered as seropositive for each indicated virus.

In this interpretation, the seroprevalence of WNV and DENV were estimated without the possibility of co-infection with ZIKV, and YFV and/or ZIKV, providing the minimum estimation of seroprevalence rates in each arbovirus.

### Viral RNA extraction and detection by reverse transcription-quantitative polymerase chain reaction (RT-qPCR)

Viral RNA was extracted from 140 μL of each serum sample using a QIAamp Viral RNA Mini Kit (Qiagen, Hilden, Germany) according to the manufacturer’s instructions. RT-qPCR was performed in a 20 μL reaction using a QuantiTect Probe RT-PCR Kit (Qiagen) [17] or a One Step PrimeScript III RT-qPCR Mix (Takara Bio, Shiga, Japan) [18]. Primers and probes specific for each arbovirus were used: WNV [19], DENV [20], YFV [21], and RVFV [22]. For ZIKV and CHIKV, the following primer and probe sets were determined after optimization: Zika_Fw (5′- GGAACTCCACACTGGAACAACA − 3′), Zika_Rv (5′-FAM-CCCTTTGCACCATCCATCTC-BHQ1–3′), ZIKA-Probe_P1 (5′-AAGGACGCACATGCCAAAAGGCAA-3′), and ZIKA-Probe_2 (5′-FAM-AAGGAYGCCCACGCCAAGAGGCAA-BHQ1–3′); CHIKV_Fw (5′-CAGTGCGGCTTCTTCAATATG-3′), CHIKV_Rv (5′-CGCATTTTGCCTTCGTAATG-3′), and CHIKV-Probe (5′-FAM-AACATCTGCACYCAAGTGTACCACAAAAGT-BHQ1–3′). The final concentrations of primers and probes for ZIKV and CHIKV were set to 0.5 μM and 0.2 μM, respectively. Those of the other viruses were set as described in the references [19–22]. RT-qPCR assays were carried out using a LightCycler 480 instrument (Roche, Basel, Switzerland) or a StepOnePlus instrument (Thermo Fisher Scientific, Waltham, MA, USA) under the following conditions: 30 min at 50 °C, 15 min at 95 °C, and 45 cycles of 15 s at 95 °C, and 60 s at 60 °C, when the QuantiTect Probe RT-PCR Kit was used; 5 min at 52 °C, 10 s at 95 °C, and 45 cycles of 5 s at 95 °C, and 35 s at 60 °C, when the One Step PrimeScript III RT-qPCR Mix was used. Data collected from the RT-qPCR assays were analysed using the software of the system. RT-qPCR assays were performed in duplicate and samples reaching threshold cycle (Ct) values under 40 were set as positive. Screening for DENV using the samples collected during 2015–2017 has been completed prior to this, which was reported previously [17].

### Statistical analyses

Statistical analyses were conducted for the samples, in which demographic information was available. The Chi-square test was used to assess the influence of demographic characteristics on the prevalence of antibodies. The student’s t-test was used to evaluate the difference in the number of arboviruses against which individuals showed seropositivity among the sexes. The one-way analysis of variance (ANOVA) was used to evaluate the difference in the number of arboviruses against which individuals showed seropositivity among the age groups; and to evaluate the difference in mean age among arbovirus-seropositive individuals. Pearson’s correlation test was used to further estimate the relationship between the number of arboviruses against which individuals showed seropositivity among the age groups. Mann-Whitney’s test was used to evaluate the difference in mean age between WNV-positive and -negative populations. All statistical analyses were conducted using GraphPad Prism version 7. A *p-*value < 0.05 was considered a statistically significant difference.

## Results

### Sample population

A total of 462 samples were collected from healthy individuals between November 2014 and January 2017 in Lambaréné, Gabon. Demographic information from 387 individuals showed that the ratio of female to male was 0.68, and the mean age was 12.2 years (range, 1–56 years). All samples were screened for IgG antibodies to the six targeted arboviruses (WNV, DENV, ZIKV, YFV, CHIKV, and RVFV) using an in-house indirect ELISA. To avoid overestimation of prevalence due to cross-reactivity within the genus *Flavivirus* (WNV, DENV, ZIKV, and YFV), cross-reactivity was examined in advance using each arbovirus seropositive control (see Additional file: Table S1). The cross-reactivity adjusted seropositivity was then determined according to the criteria described in the Methods section.

### Seroprevalence of WNV, DENV, ZIKV, YFV, CHIKV, and RVFV

The prevalence of antibodies is summarized in Table 1. The overall prevalence of antibodies to each arbovirus in the 387 individuals with demographic information was as follows: WNV, 25.3% (98/387); DENV, 20.4% (79/387); ZIKV, 40.3% (156/387); YFV, 60.7% (235/387); CHIKV, 61.2% (237/387); and RVFV, 14.3% (55/387). A similar prevalence was observed in the 462 individuals tested, including the sample whose demographic information was unavailable. There was no significant difference in the prevalence between the male and female populations. Focusing on age, the prevalence of antibodies to YFV and RVFV increased gradually with age. Moreover, seropositivity against ZIKV and CHIKV showed a clear increase in individuals aged > 17 years compared with those aged 1–2 years (*p* < 0.001). In contrast, the prevalence of antibodies to WNV was comparable among individuals in the age groups of 1–2 years, 3–11 years, and > 17 years, and showed a slight decrease in those aged 12–17 years (*p* = 0.55). Similarly, the prevalence of antibodies to DENV was comparable among individuals in the age groups of 1–2 years, 3–11 years, and 12–17 years, and significantly declined in individuals aged > 17 years (*p* = 0.027). These results indicate that WNV, DENV, ZIKV, CHIKV, and RVFV have different circulation patterns around the study area. This observation was also supported by the results presenting different prevalence in the collection year; for example, a statistically significant decreasing trend was observed in CHIKV (2014, 86.8%; 2015, 66.2%; 2016, 53.3%; 2017, 58.8%; *p* = 0.0004), whereas an increasing trend was observed in DENV (2014, 10.5%; 2015, 23.1%; 2016, 18.9%; 2017, 29.4%; *p* = 0.25). Because of the free yellow fever-vaccine program available in the country, the prevalence of antibodies to YFV might reflect vaccine-immunization coverage, but not the actual virus infection. The prevalence of antibodies to YFV in terms of vaccine-immunization coverage is presented in the “Discussion” section below.

**Table 1:** Prevalence of antibodies to the six arboviruses of public health concerns in Gabon

Characteristics	Total No.	WNV	DENV	ZIKV	YFV	CHIKV	RVFV
Overall prevalence							
*N* (% [95% CI])	462^a^	111 (24.0 [20.1–27.9])	103 (22.3 [18.5–26.1])	179 (38.7 [34.3–43.2])	275 (59.5 [55.0–64.0])	278 (60.2 [55.7–64.6])	67 (14.5 [11.3–17.7])
	387^b^	98 (25.3 [21.0–29.6])	79 (20.4 [16.4–24.4	156 (40.3 [35.4–45.2])	235 (60.7 [55.9–65.6])	237 (61.2 [56.4–66.1])	55 (14.3 [10.7–17.7])
Sex							
Female	157	35 (22.3 [18.2–26.4])	39 (24.8 [20.5–29.1])	60 (38.2 [33.4–43.1])	92 (58.6 [53.7–63.5])	91 (58.0 [53.0–62.9])	20 (12.7 [9.4–16.1])
Male	230	63 (27.4 [23.0–31.8])	40 (17.4 [13.6–21.2])	96 (41.7 [36.8–46.7])	143 (62.2 [57.3–67.0])	146 (63.5 [58.7–68.3])	35 (15.2 [11.6–18.8])
Age							
1–2 years	79	23 (29.1 [24.6–33.6])	21 (26.6 [22.2–31.0])	23 (29.1 [24.6–33.6])	42 (54.2 [48.2–58.1])	32 (40.5 [35.6–45.4])	10 (12.7 [9.3–16.0])
3–11 years	140	36 (25.7 [21.3–30.1])	31 (22.1 [18.0–26.2])	43 (30.7 [26.1–35.3])	82 (58.6 [53.7–63.5])	67 (47.9 [42.9–52.8])	18 (12.9 [9.5–16.2])
12–17 years	28	3 (10.7 [7.6–13.8])	7 (25.0 [20.7–29.3])	13 (46.4 [41.5–51.4])	17 (60.7 [55.8–65.6])	22 (78.6 [74.5–82.7])	4 (14.3 [10.8–17.8])
> 17 years	140	36 (25.7 [21.3–30.1])	20 (14.3 [10.8–17.8])	77 (55.0 [50.0–60.0])	94 (67.1 [62.5–71.8])	116 (82.9 [79.1–86.6])	23 (16.4 [12.7–20.1])
Collection year							
2014	38	13 (34.2 [29.5–38.9])	4 (10.5 [7.5–13.6])	20 (52.6 [47.7–57.6])	28 (73.7 [69.3–78.1])	33 (86.8 [83.5–90.2])	4 (10.5 [7.5–13.6])
2015	130	33 (25.4 [21.0–29.7])	30 (23.1 [18.9–27.3])	58 (44.6 [39.7–49.6])	75 (57.7 [52.8–62.6])	86 (66.2 [61.4–70.9])	20 (15.4 [11.8–19.0])
2016	185	45 (24.3 [20.0–28.6])	35 (18.9 [15.0–22.8])	60 (32.4 [27.8–37.1])	110 (59.5 [54.6–64.4])	98 (53.0 [48.0–57.9])	28 (15.1 [11.6–18.7])
2017	34	7 (20.6 [16.6–24.6])	10 (29.4 [24.9–34.0])	18 (52.9 [48.0–57.9])	22 (64.7 (59.9–69.5))	20 (58.8 [53.9–63.7])	3 (8.8 [6.0–11.6])

*WNV* West Nile virus, *DENV* dengue virus, *ZIKV* Zika virus, *YFV* yellow fever virus, *CHIKV* chikungunya virus, *RVFV* Rift Valley fever virus, CI confidence interval

^a^ All tested samples regardless of demographic information

^b^ Samples with demographic information available

### Number of seropositive-arboviruses in individuals

**Fig. 2:**
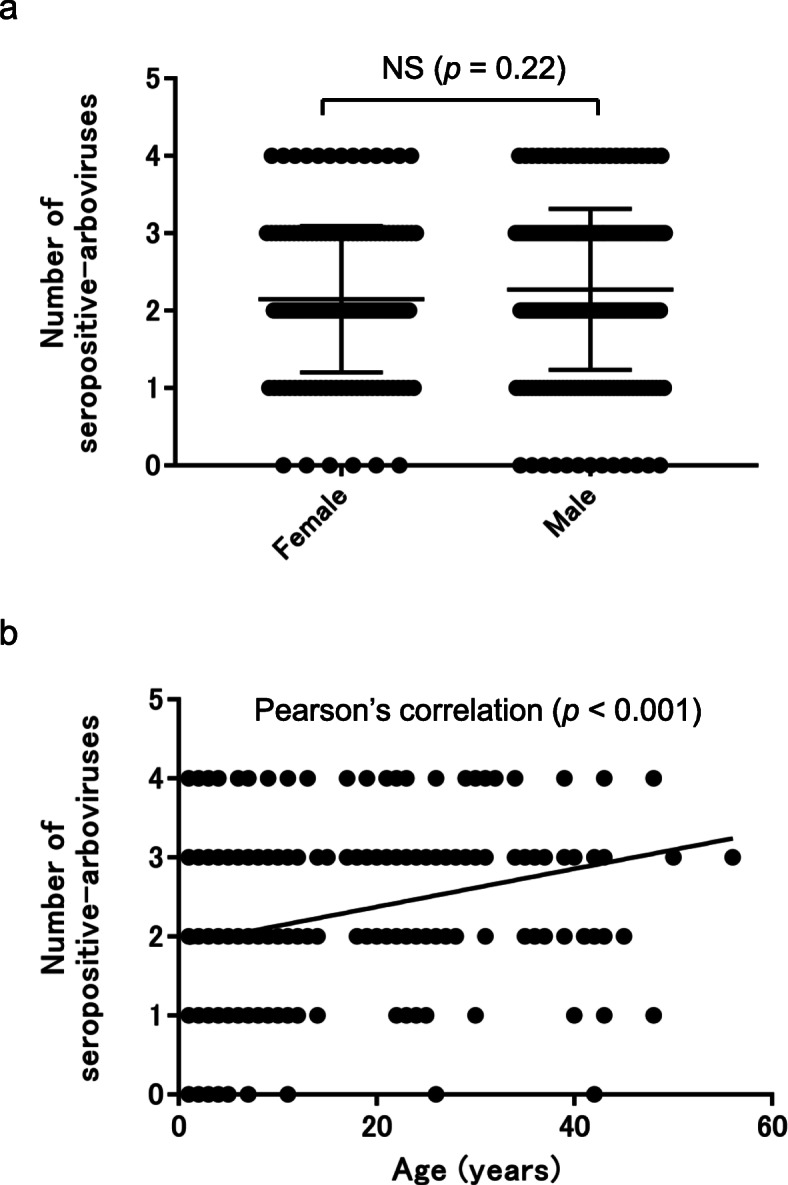
Number of seropositive-arboviruses. NS: Not significance

To evaluate the risk of arbovirus infections in the country, the number of arboviruses against which individuals showed seropositivity was analysed. The results indicated that there was no significant difference between the male and female populations (Fig. 2a). Individuals aged ≥3 years had antibodies to two or more arboviruses, and even those aged 1–2 years had antibodies to nearly two arboviruses. Individuals aged > 17 years had antibodies to more arboviruses than those aged ≤11 years, with significant differences (*p* < 0.001) (Additional file 2: Table S2). A Pearson’s correlation test indicated a positive correlation between age and the number of arboviruses against which an individual showed seropositivity (r = 0.29; *p* < 0.001) (Fig. 2b), which means that the residents of Gabon displayed an increase in their immunity to arboviruses across their lifespans. These results provide evidence for the simultaneous circulation of several arboviruses and exposure of the population to these viruses in the early stages of life in Gabon.

### Mean age of each arbovirus-seropositive individual

**Fig. 3:**
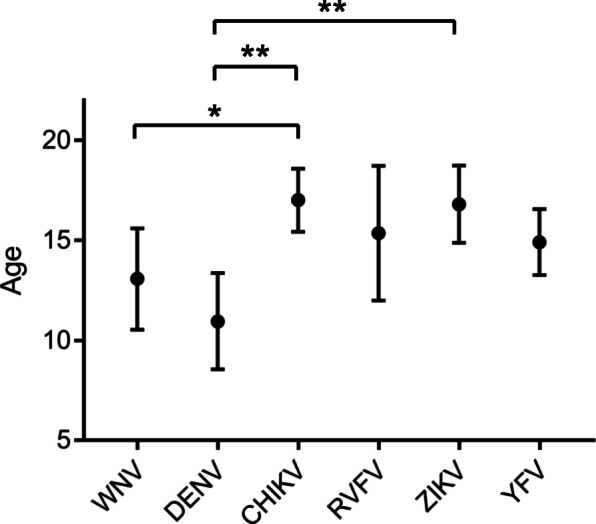
Comparison of mean age of each arbovirus-seropositive individual. Black circle: Mean; Error bar: 95% CI; *: *p* < 0.05; **: *p* < 0.01

To estimate the period of the arbovirus circulation in the country, the mean age of each arbovirus-seropositive individual was further analysed (Fig. 3). We found mean ages ranging from 11.0 to 17.0 years among the arboviruses we tested. The mean age in WNV-seropositive individuals (aged 13.1 years) was significantly lower than in CHIKV-seropositive individuals (aged 17.0 years, *p* = 0.031), and the mean age in DENV-seropositive individuals (aged 11.0 years) was significantly lower than in both ZIKV-seropositive individuals (aged 16.8 years, *p* = 0.0044) and in CHIKV-seropositive individuals (aged 17.0 years, *p* = 0.0010). These results indicate that, in Gabon, ZIKV and CHIKV had probably circulated in the past, but not recently, whereas WNV and DENV have circulated recently or is currently circulating. This result appears to be consistent with the prevalence of antibodies to these viruses shown in Table 1; the prevalence was highest in individuals aged > 17 years in ZIKV and CHIKV, whereas in those aged 1–2 years the highest prevalence was of WNV and DENV.

### Detection of WNV and DENV genomes in febrile patients

In addition, at the same time as the serological survey, we continued to investigate the current situation of the six targeted arboviruses using serum samples of patients with fever. Demographic information of the samples showed that the ratio of female to male was 0.96, and the mean age was 10.3 years (range, 1–82 years).

A total of 1189 samples were screened for WNV, DENV, ZIKV, YFV, CHIKV, and RVFV by RT-qPCR. In Gabon, we detected WNV in three samples collected in 2020 (Table 2). We detected DENV-3 in 17 samples during 2016–2017, as we previously reported [17], but no DENV was detected in samples collected in 2020. All WNV-positive samples were of individuals aged ≤5 years and a comparison of the mean age for all samples showed that the WNV-positive population was younger than the negative population, while the DENV-positive population was older with a significant difference, as described previously [17]. The RT-qPCR results revealed that WNV and DENV are currently circulating in Gabon, which is consistent with the prediction from the serological surveys.

**Table 2:** Demographic and laboratory data of patients infected with WNV

Sample ID	Age (years)	Sex	Ct value	Collection Date	Mean age (95% CI)		*p*-value
					Positive-population	Negative-population	
WNV					2.3 (−1.5–6.1)	10.2 (9.4–11.0)	0.14
01	2	M	35.8	25 Feb 2020			
02	4	M	34.3	9 Mar 2020			
03	1	M	37.0	16 Mar 2020			

*WNV* West Nile virus, *CI* confidence interval

## Discussion

In Gabon, at least 24 genomic and serological data on arbovirus infections have been reported; however, available information is still limited (Additional file 3: Table S3). This is a comprehensive surveillance study of arboviruses, which are public health concerns in Gabon, and it has revealed the latest seroprevalence state of the six arboviruses (WNV, DENV, ZIKV, YFV, CHIKV, and RVFV) of the study area in healthy individuals collected during 2014–2017. Notably, WNV and DENV genomes were detected in febrile patients, as our serological analyses predicted an increased potential risk of infection with these two viruses. Here, the historical scenario and future risk of arbovirus infections in Gabon are further discussed.

### WNV

The first report of WNV in Gabon can be dated back between 2002 and 2005, a report showing seropositivity in horses [23]. A serological survey conducted between 2005 and 2008 showed a 23.7% prevalence in humans in rainforest regions (including Lambaréné) in Gabon [24]. In 2009, the first and only human case diagnosed the WNV infection by molecular assay was recorded in Gabon [25]. Our study updated the information on the seroprevalence of WNV (25.3%) and found that seroprevalence seems to be stable (approximately 25%) in the region for over 10 years. Furthermore, after our serological survey was conducted during 2014–2017, patients presenting with fever due to WNV infection were found in 2020, which are the first cases to detect WNV genomes with information of clinical pictures (Additional file 4: Table S4) in the country. A systematic review demonstrated that WNV infection is endemic throughout sub-Saharan Africa [26]. Nevertheless, reports of WNV infections have not been available from the neighbouring countries such as Cameroon, Equatorial Guinea, and the Republic of the Congo, and further, there have been no reports of major burdens of WNV in Africa for over 15 years since the period when large outbreaks occurred in South Africa, the Democratic Republic of the Congo, and Sudan [27–29]. WNV has been circulating at least for two decades and detection of the viral genomes in this study revealed a re-emergence of WNV in Gabon; therefore, careful monitoring is required to prevent its spread.

### DENV

Gabon experienced two dengue outbreaks in 2007 and 2010; these outbreaks were associated with simultaneous CHIKV infections [14, 16]. Although the seroprevalence of DENV (20.4%) was much lower than that of CHIKV (61.2%), the results of this study were consistent with the seroprevalence found in patients during 2007–2010 (DENV, 8%; CHIKV, 40%) [16]. The current seroprevalence in Gabon was slightly higher than the pooled seroprevalence in Africa (15.6%), which varied from 8.0% in Southern Africa to 38.6% in Central Africa [30]. Our findings suggest that in the most recent circulation of DENV in Lambaréné, the mean age of DENV-seropositive individuals was the youngest among the tested viruses. In fact, the DENV-3 genome was detected in febrile patients in 2016–2017, as we previously reported [17], but no new patients were found in 2020. Until now, DENV-1, − 2, and − 3 genomes have been detected in Lambaréné, leading to the possibility of secondary infections with other serotypes, which may cause severe symptoms [31]. The severity of DENV infection due to pre-existing antibodies to ZIKV was also documented (and vice versa) [32]. Although the serotype of DENV were undistinguishable in our ELISA, more attention to DENV infections with an increasing potential risk of severe cases should be paid in Gabon.

### ZIKV

A history of ZIKV in Gabon began between 1970 and the 1980s, when antibodies to ZIKV were detected in both humans and monkeys from the south-eastern parts [33, 34]. After over 30 years, the ZIKV genome was detected in humans in 2007 in Cocobeach, in the north-western part of Gabon, and in *Aedes albopictus* mosquitos in 2007 and 2010 in Libreville (the capital city), which was the first documentation of an outbreak in Africa (Fig. 1) [35]. Meanwhile, neither detection of the viral genomes nor antibodies have been reported in Lambaréné so far. This study yielded the first serological evidence for the existence of ZIKV in Lambaréné. The seroprevalence decreased with age, indicating that ZIKV is presently circulating with a decreasing risk of infections. At present, ZIKV-seropositivity has been recognized in at least 25 countries in Africa [36]. Although the strains identified in Gabon and other African countries belong to the African lineage only for a long time, the introduction of Asian lineage strains, which are well-known to be associated with severe birth defects, has recently been reported in Angola with microcephaly cases [37]. Even though the risk of ZIKV infection apparently drop now, the pathogenicity of the African lineage and a new introduction of Asian lineage strains to the country are still to be elucidated, and thus continuous surveillance will be important in clinical settings, especially regarding significant severe birth defects.

### YFV

Despite the free yellow fever-vaccine program available in Gabon, the prevalence of antibodies seemed to be insufficient to suppress the spread of the disease. A past report on serological survey between 1970 and the 1980s presented that more than 80% of adults had neutralizing antibodies for YFV as consequence of a mass vaccination campaign in Gabon [33, 34]. After over 30 years with a lack of information, the World Health Organization (WHO) and United Nations Children’s Fund (UNICEF) have started to estimate the annual immunisation coverage since 2003, using the limited data reported by the government. In the estimation, the prevalence of the antibody showed a remarkable increase during 2003–2007, but since then a slight reduction was observed [38]. The estimated prevalence during 2014–2017 was similar to that in our result (60.7%), enhancing the reliability of the results in this study. Compared to other yellow fever-endemic countries in Africa, the immunisation coverage in Gabon was lower than the average (Additional file 5: Table S5). Mali and Nigeria, where immunisation coverages were similar to that of Gabon, have recently experienced yellow fever outbreaks, suggesting that the current immunisation coverage in Gabon would be insufficient for herd immunity. To protect residents of Gabon from the threat of YFV infection, it is necessary to accurately understand the vaccination status through regular serological surveys and to evaluate the effect of the national vaccine program.

### CHIKV

In contrast to DENV, the seroprevalence of CHIKV infections increased with age, and the mean age of CHIKV-seropositive individuals was the oldest among the targeted arboviruses, indicating that CHIKV is presently circulating with a decreasing risk of infections, such as ZIKV. Similar to other countries such as Kenya, that experienced CHIKV outbreaks [39], Gabonese adults (aged > 17 years) who presented with a high seroprevalence of CHIKV (82.9%) seem to be at low risk of CHIKV infection due to herd immunity. However, as shown in the Republic of the Congo, where the seroprevalence was 34.4% before the first CHIKV outbreak in 2011 and the second outbreak occurred in 2019 [40, 41], Gabonese children (aged ≤11 years) who presented with a low seroprevalence (40.5–47.9%) are at a relatively high risk of a new outbreak of the virus.

Here, we propose a hypothesis to explain the different circulation patterns of DENV, ZIKV, and CHIKV in Gabon with a focus on vectors. These viruses showed unique vector selections in a virus-specific manner during outbreaks in the country [14, 15]. ZIKV and CHIKV were predominant in *A. albopictus*, whereas DENV was predominant in *A. aegypti*. Compared to *A. albopictus*, recent studies demonstrated that *A. aegypti* preferentially used artificial cavities or containers for the breeding, and spread of *A. aegypti*, was significantly related to human activity [42, 43]. Considering the population increase in Lambaréné in this decade, *A. aegypti* could adapt to a new environment with increasing human activity more rapidly than *A. albopictus*. Consequently, the increasing number of *A. aegypti* causes a recent increase in DENV infections in Lambaréné. Investigation of the distribution and virus possession of the vectors will provide a better understanding of the dynamics of these virus circulations in the area.

### RVFV

Compared to a previous serological survey conducted during 2005–2008, the seroprevalence of RVFV has increased in Gabon (from 2.9 to 14.3%) [44]. Rift Valley fever outbreaks have been reported in sub-Saharan Africa and North Africa since its first discovery in Kenya in 1931 [45], and its seroprevalence ranges from 0 to 77.0% throughout Africa [46]. The current seroprevalence (14.3%) in Gabon was similar to that in the countries that experienced its outbreak, such as Kenya and South Africa [47–49]. Although clinical case of RVF has not been reported in Gabon, RVFV or unidentified RVF-like viruses appear to be silently circulating in Gabon and there might be a risk of an outbreak at the equivalent level to the endemic areas. Focusing on the viral transmission cycle between mosquitoes (*Aedes* and *Culex* spp.), humans and animals (e.g., sheep, goats, cattle, and camel), seropositivity was documented in sheep and goats in 2014 in the country [50]. A systematic review demonstrated that there was no significant difference in seroprevalence during outbreaks compared to interepidemic periods in human populations, but this was not the case in sheep and goats [46]. Monitoring the temporal change of seroprevalence in the animals would provide an additional clue to predict the emergence of RVFV.

### Limitations

In this study, seroprevalence for six arboviruses of public health concern in Gabon was examined by ELISA. However, we cannot completely exclude the possibilities that the results include the samples positive for other related flaviviruses, phleboviruses or alphaviruses, such as Usutu virus and O’nyong-nyong virus, which might cause cross-reaction with the viruses examined in this study, although human infections with such viruses have not reported in this area so far and the result is reasonable in accordance with the WHO-UNICEF estimation [38], nevertheless, the results would be feasible to reveal the infection risk of arboviruses in Gabon.

## Conclusion

This study demonstrated the latest serological state and the potential risk of arbovirus infections in Gabon. Furthermore, the study revealed the re-emergence of WNV as well as DENV in the country. This information will contribute to a rapid response to arbovirus infections, thereby preventing future spread in the country.

## Supplementary Information

**Additional file 1: Table S1.** OD value examined to determine cross-reactivity within the genus Flavivirus

**Additional file 2: Table S2.** The number of people infected with different arboviruses

**Additional file 3: Table S3.** Studies on the arbovirus infections which are public health concerns in Gabon

**Additional file 4: Table S4.** Clinical pictures of patients infected with WNV

**Additional file 5: Table S5.** Coverage of yellow fever-vaccine and recent report in African countries at risk for yellow fever epidemic

## Data Availability

The datasets used and/or analysed during the current study are available from the corresponding author upon reasonable request.

## Abbreviations

WNV: West Nile virus
DENV: Dengue virus
ZIKV: Zika virus
YFV: Yellow fever virus
CHIKV: Chikungunya virus
RVFV: Rift Valley fever virus
CERMEL: Centre de Recherches Médicales de Lambaréné
ELISA: Enzyme-linked immunosorbent assay
NS1: Non-structural protein 1
E1: Envelope protein 1
NP: Nucleoprotein
PBS: Phosphate-buffered saline PBS
TMB: 3,3′,5,5′-tetramethylbenzidine
CI: Confidence interval
RT-qPCR: Reverse transcription-quantitative polymerase chain reaction
WHO: World Health Organization
UNICEF: United Nations Children’s Fund
